# Antibody Responses in SARS-CoV-2-Exposed and/or Vaccinated Individuals Target Conserved Epitopes from Multiple CoV-2 Antigens

**DOI:** 10.3390/ijms25189814

**Published:** 2024-09-11

**Authors:** David Yao, Raj S. Patel, Adrien Lam, Quarshie Glover, Cindy Srinivasan, Alex Herchen, Bruce Ritchie, Babita Agrawal

**Affiliations:** 1 Department of Surgery, Faculty of Medicine and Dentistry, College of Health Sciences, University of Alberta, Edmonton, AB T6G 2R3, Canada; dtyao@ualberta.ca (D.Y.); rsp@ualberta.ca (R.S.P.); adrien2@ualberta.ca (A.L.); 2 Department of Medicine, Faculty of Medicine and Dentistry, College of Health Sciences, University of Alberta, Edmonton, AB T6G 2R3, Canada; gq.quarshie@yahoo.com (Q.G.); csriniva@ualberta.ca (C.S.); aherchen@ualberta.ca (A.H.); bruce.ritchie@ualberta.ca (B.R.)

**Keywords:** SARS-CoV-2, antibody, conserved epitopes, vaccine

## Abstract

There is a need to investigate novel strategies in order to create an effective, broadly protective vaccine for current and future severe acute respiratory syndrome coronavirus 2 (SARS-CoV-2) outbreaks. The currently available vaccines demonstrate compromised efficacy against emerging SARS-CoV-2 variants of concern (VOCs), short-lived immunity, and susceptibility to immune imprinting due to frequent boosting practices. In this study, we examined the specificity of cross-reactive IgG antibody responses in mRNA-vaccinated, AstraZeneca-vaccinated, and unvaccinated donors to identify potentially conserved, cross-reactive epitopes to target in order to create a broadly protective SARS-CoV-2 vaccine. Our study provides evidence for cross-reactive IgG antibodies specific to eight different spike (S) variants. Furthermore, the specificities of these cross-variant IgG antibody titers were associated to some extent with spike S_1_- and S_2_-subunit-derived epitopes P_1_ and P_2_, respectively. In addition, nucleocapsid (N)- and membrane (M)-specific IgG antibody titers correlated with N- and M-derived epitopes conserved across beta-CoVs, P_3–7_. This study reveals conserved epitopes of viral antigens, targeted by natural and/or vaccine-induced human immunity, for future designs of next-generation COVID-19 vaccines.

## 1. Introduction

The global outbreak of the SARS-CoV-2 has infected >775 million people and caused >7 million deaths worldwide [1]. As of June 2024, a total of 5.47 billion vaccine doses of first-generation COVID-19 vaccines (Pfizer/BioNTech-BNT162b2, Moderna-mRNA-1273, AstraZeneca-ChAdOx1nCov-19, Johnson & Johnson-Ad26.COV2-S) and the newly approved bivalent boosters have been administered [1]. Thus far, vaccination has been the most effective strategy against SARS-CoV-2, as these vaccine efforts have mitigated the risk of severe disease outcomes, hospitalizations, and mortalities [2]. However, vaccine-induced immunity appears to be short-lived and wanes over time [3]. Furthermore, repeated booster shots with the latest spike variants are susceptible to back-boosting immunity as well as antigenic sin, which involves imprinting antibody responses against the original Wuhan-Hu-1 strain and limiting the generation of de novo antibody responses against new SARS-CoV-2 variants [4,5]. Taking account of these limitations, next-generation vaccines should target conserved and broadly protective epitopes while avoiding the pooling of S-proteins from different SARS-CoV-2 VOCs to establish cross-reactivity [6].

Exploring heterologous immunity could provide valuable insights for addressing the limitations of current vaccines. Heterologous immunity refers to the immune responses against one pathogen providing cross-protection against a different pathogen. This phenomenon partly relies on the cross-reactivity of adaptive immune responses against conserved epitopes shared between different pathogens [7]. While heterologous immunity has been well documented in murine models, its application to SARS-CoV-2, in identifying functionally conserved, cross-reactive epitopes, is poorly understood. The humoral arm of the immune system, which involves the production of neutralizing antibodies, has been associated with protection from SARS-CoV-2 infections [8]. Moreover, N- and M-specific antibody responses have shown protective effects against disease outcomes and have been found to reduce viral shedding [9,10]. Therefore, investigating cross-reactive antibodies capable of recognizing conserved epitopes against novel SARS-CoV-2 VOCs in individuals previously infected with SARS-CoV-2 and/or vaccinated with first-generation COVID-19 vaccines can facilitate the development of an effective vaccine that limits back-boosting, immune imprinting, and the antigenic sin phenomena, while promoting the generation of de novo antibody responses. Considering the high sequence homology of SARS-CoV-2 with other human CoVs, and the presence of highly conserved regions within SARS-CoV-2 structural proteins (S, N, and M), our study aims to support the development of vaccines that offer broader and longer-lasting protection. All in all, by understanding cross-reactive antibodies generated by natural infection and/or vaccination, we can better design vaccines to combat current and future SARS-CoV-2 variants and other emerging CoVs.

Our study investigates the specificity of cross-reactive IgG antibodies in individuals who have been infected and/or vaccinated with the first-generation COVID-19 vaccines. We hypothesize that understanding the specificity of cross-reactive antibody responses against conserved epitopes within the SARS-CoV-2 S (P_1_/P_2_), N (P_3_/P_4_/P_5_), and M (P_6_/P_7_) proteins can provide a basis for designing a pan-coronavirus vaccine candidate (Table 1). P_1–7_ epitopes were selected based on major histocompatibility complex (MHC) class I and II binding predictions, CD4^+^/CD8^+^ T cell and B cell epitope predictions, and conservativity scoring among CoVs (Table 1) [10,11,12,13,14,15,16,17,18,19,20,21]. Furthermore, we determined the IgG response against seven lipopeptide constructs (LP_1–7_), incorporating P_1–7_ epitopes conjugated with a palmitoyl moiety, to explore the potential immunogenicity of lipopeptide-based subunit vaccines with respect to human humoral immunity [22]. Ultimately, this work lays the foundation for the creation of a vaccine capable of providing long-term, cross-reactive immunity against a broad spectrum of CoVs, addressing the challenges posed by the continual evolution of SARS-CoV-2 and related viruses.

## 2. Results

### 2.1. Plasma Donor Demographics

In this study, plasma samples from a total of 41 individuals were used to assess IgG antibody titers from mRNA-vaccinated (n = 15), AstraZeneca-vaccinated (n = 11), and unvaccinated (n = 15) donors. Samples were collected between July 2020 and Jan 2022 (Table 2). Among the mRNA-vaccinated donors, there were 11 females (73.3%) and 4 males (26.7%), with a median age of 25 years. Moreover, the mRNA-vaccinated cohort had no donors reported with COVID-19 infections. The AstraZeneca-vaccinated donors had an approximately equal proportion of six females (54.5%) and five males (45.5%), with a median age of 58 years and six donors with reported COVID-19 infections. The unvaccinated donors had a median age of 42 years, and this sample was composed of 6 females (40.0%) and 9 males (60.0%), with 10 donors reported to be infected with COVID-19.

### 2.2. mRNA and AstraZeneca-Vaccinated Donors Generated Cross-Reactive IgG Antibody Titers against Multiple SARS-CoV-2 Spike Variants and P_1_/P_2_ Spike Epitopes

**Figure 1:**
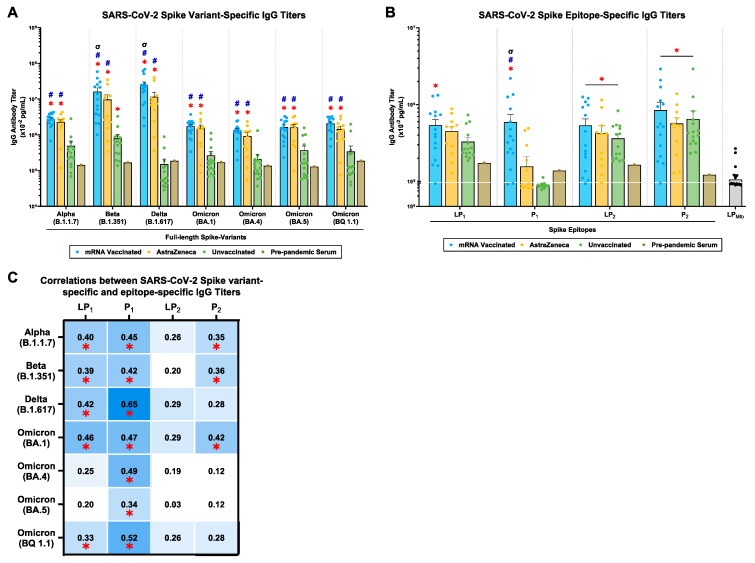
mRNA and AstraZeneca-vaccinated donors generated cross-reactive SARS-CoV-2 spike variant-specific IgG antibody titers. Plasma samples from mRNA-vaccinated (n = 15; blue), AstraZeneca-vaccinated (n = 11; yellow), and unvaccinated (n = 15; green) donors were assayed for IgG antibodies against (**A**) the SARS-CoV-2 S-proteins of the Alpha (B.1.1.7), Beta (B.1.351), Delta (B.1.617), and Omicron (BA.1, BA.4, BA.5, BQ1.1) variants, and (**B**) S-derived lipopeptides (LP_1_ and LP_2_) and peptides (P_1_ and P_2_). Pre-pandemic serum and LP_Mtb_ were used as controls. Bars represent mean ± SEM and each point represents an individual donor. Statistical significance was determined using a two-way ANOVA, followed by Tukey’s post hoc test. *, #, and σ indicates significant differences (*p* ≤ 0.05) between the pre-pandemic serum, the unvaccinated donors, and the mRNA vs. AstraZeneca donors, respectively. (**C**) Correlations between SARS-CoV-2 spike variant-specific and epitope-specific IgG antibody titers were conducted. Spearman’s R-values (black) and significant correlations (*p* ≤ 0.05; *) are indicated.

Plasma samples from mRNA-vaccinated, AstraZeneca-vaccinated, and unvaccinated donors were evaluated for IgG antibodies against the S-protein of the SARS-CoV-2 Alpha (B.1.1.7), Beta (B.1.351), Delta (B.1.617), and Omicron (BA.1, BA.4, BA.5, BQ1.1) variants (Figure 1). mRNA and AstraZeneca-vaccinated donors generated significantly higher cross-reactive IgG antibody titers across all SARS-CoV-2 variants compared to the unvaccinated donors and pre-pandemic serum (Figure 1A). Moreover, the mRNA-vaccinated group showed significantly higher IgG antibody titers against the Beta (B.1.351) and Delta (B.1.617) variants compared to the AstraZeneca-vaccinated group (Figure 1A). Notably, in the unvaccinated group, there were detectable levels of IgG antibody titers across all SARS-CoV-2 variants, with IgG antibody titers against the Beta (B.1.351) variants showing significance compared to pre-pandemic serum (Figure 1A). Also, there was a decline in IgG antibody titers against the latest Omicron (BA.1, BA.4, BA.5, BQ1.1) variants compared to the earlier Alpha (B.1.1.7), Beta (B.1.351), and Delta (B.1.617) variants. Overall, mRNA and AstraZeneca-vaccinated donors generated a cross-reactive IgG antibody response against the SARS-CoV-2 Alpha (B.1.1.7), Beta (B.1.351), Delta (B.1.617), and Omicron (BA.1, BA.4, BA.5, BQ1.1) variants.

Next, plasma samples were assayed for IgG antibody titers against spike lipopeptides (LP_1_ and LP_2_), and their peptide counterparts (P_1_ and P_2_). LP_1_-specific IgG antibody titers were highest in the mRNA-vaccinated group, followed by the AstraZeneca-vaccinated and unvaccinated groups (Figure 1B). Similarly, P_1_-specific IgG antibody titers were significantly higher in the mRNA-vaccinated group compared to the AstraZeneca, unvaccinated, and pre-pandemic serum (Figure 1B). Furthermore, LP_2_/P_2_-specific IgG antibody titers were significantly higher in the mRNA, AstraZeneca, and unvaccinated groups, compared to pre-pandemic serum (Figure 1B). As a control, all donor plasma samples were tested against a *Mycobacterium tuberculosis* (LP_Mtb_)-derived lipopeptide to differentiate the peptide-specific binding of IgG antibody titers, which were tested against LP_1_ and LP_2_, and not towards the lipid tail of the lipopeptides. LP_Mtb_-specific IgG antibody titers showed titers even lower than those in the pre-pandemic serum (Figure 1B). Altogether, plasma IgG antibodies were detected against all S-derived lipopeptides (LP_1_ and LP_2_) and peptides (P_1_ and P_2_), with mRNA-vaccinated groups showing the highest titer, followed by AstraZeneca-vaccinated and unvaccinated donors. 

Moreover, correlation studies showed that LP_1_-specific IgG titers significantly correlated with IgG titers against the S-protein of the SARS-CoV-2 Alpha (B.1.1.7), Beta (B.1.351), Delta (B.1.617), and Omicron (BA.1, BQ1.1) variants (Figure 1C). P_1_-specific IgG titers significantly correlated with all tested SARS-CoV-2 variants (Figure 1C). However, LP_2_-specific IgG titers did not correlate significantly with any of the tested SARS-CoV-2 variants (Figure 1C). Finally, P_2_-specific IgG titers showed moderate correlations with SARS-CoV-2 Alpha (B.1.1.7), Beta (B.1.351), and Omicron (BA.1) variants (Figure 1C). 

In conclusion, mRNA-vaccinated, AstraZeneca-vaccinated, and unvaccinated donors induced cross-reactive IgG antibody titers against multiple SARS-CoV-2 spike variants, which correlated with P_1_ and P_2_ epitope specificity (Figure 1).

### 2.3. Competitive Inhibition with P_1_ and P_2_ Epitopes Reduced Cross-Reactive IgG Antibody Binding to Multiple SARS-CoV-2 Spike Variants in mRNA, AstraZeneca, and Unvaccinated Donors

**Figure 2:**
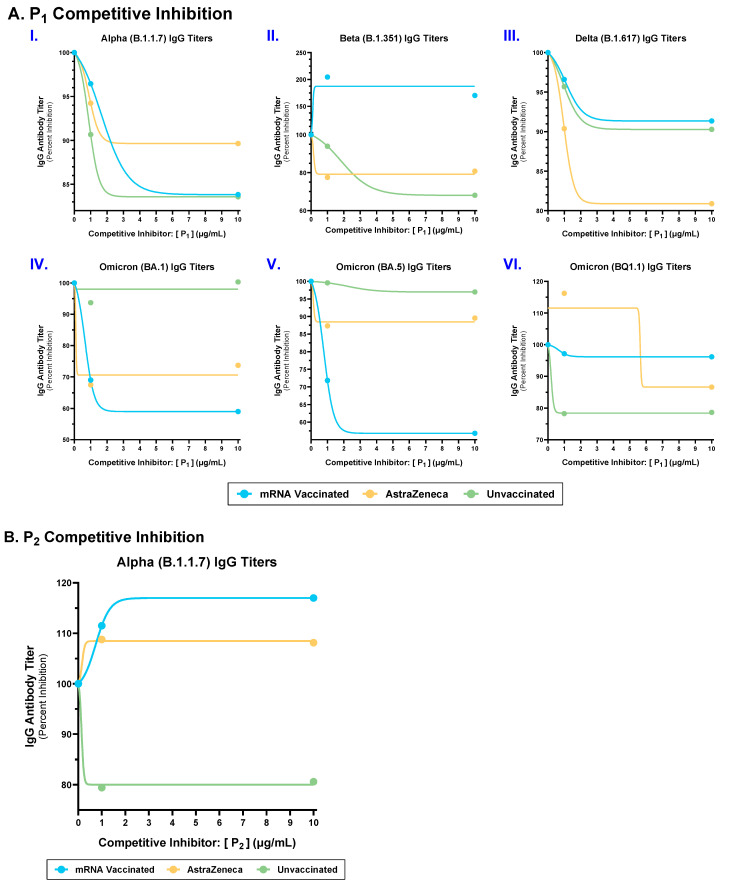
Competitive inhibition of SARS-CoV-2 spike variant-specific IgG antibody titers by P_1_ and P_2_ epitopes in mRNA-vaccinated, AstraZeneca-vaccinated, and unvaccinated donors. Percent inhibition of IgG antibody titers in mRNA-vaccinated (n = 3–5; blue), AstraZeneca-vaccinated (n = 3–5; yellow), and unvaccinated (n = 3–5; green) donors, after competitively inhibiting (**A**) P_1_ and (**B**) P_2_ epitopes against SARS-CoV-2 (**I**) Alpha (B.1.1.7), (**II**) Beta (B.1.351), (**III**) Delta (B.1.617), and (**IV**–**VI**) Omicron (BA.1, BA.5, BQ1.1) variants. Each point represents the mean percent inhibition of at least 3 donors (n = 3–5). Inhibition curves were generated by fitting data points to a sigmoidal dose–response curve using GraphPad Prism.

To determine the specificity of cross-reactive SARS-CoV-2 spike variant-specific IgG antibody titers, we carried out competitive inhibition experiments using increasing concentrations of P_1_ and P_2_ epitopes and measured IgG binding to the SARS-CoV-2 Alpha (B.1.1.7), Beta (B.1.351), Delta (B.1.617), and Omicron (BA.1, BA.5, BQ1.1) variants (Figure 2). P_1_ epitope has acquired mutations in variants; however, it is structurally conserved among SARS-CoV-2 VOCs and is predicted to be a discontinuous B cell epitope, whereas P_2_ is sequentially and structurally conserved among CoVs [13,16,17,20,21]. In the mRNA-vaccinated group, competitive inhibition with P_1_ epitope partially reduced IgG binding to the SARS-CoV-2 Alpha (B.1.1.7), Delta (B.1.617), Omicron (BA.1), and Omicron (BA.5) variants (Figure 2(AI,III–V)). In contrast, competitive interactions with P_1_ epitope enhanced IgG binding to the SARS-CoV-2 Beta (B.1.351) variant in the mRNA-vaccinated group (Figure 2(AII)), whereas no reduction in IgG binding was observed against the SARS-CoV-2 Omicron (BQ1.1) variants (Figure 2(AVI)). Next, AstraZeneca-vaccinated donors showed a partial reduction in IgG binding against all tested SARS-CoV-2 variants (Figure 2(AI–VI)). Furthermore, the unvaccinated groups showed a reduction in IgG binding against the SARS-CoV-2 Alpha (B.1.1.7), Beta (B.1.351), Delta (B.1.617), and Omicron (BQ1.1) variants (Figure 2(AI–III,VI)). However, there was no reduction in IgG binding against the SARS-CoV-2 Omicron (BA.1), and Omicron (BA.5) variants (Figure 2(AIV,V)).

Considering P_2_ is a sequentially conserved epitope among SARS-CoV-2/CoV, we competitively inhibited plasma samples with the P_2_ epitope only against the SARS-CoV-2 Alpha (B.1.1.7) variant [23]. The unvaccinated group showed a 20% reduction in IgG binding, whereas the mRNA and AstraZeneca-vaccinated groups showed an increase in IgG binding against the SARS-CoV-2 Alpha (B.1.1.7) variant by 17% and 4%, respectively (Figure 2B). 

In conclusion, these results suggest that in the human IgG repertoire against spike antigens, at least a fraction of antibodies are targeted against P_1_- and P_2_-conserved epitopes.

### 2.4. mRNA, AstraZeneca, and Unvaccinated Donors Showed IgG Antibody Titers against the SARS-CoV-2 N (Protein), N-Derived Lipopeptides (LP_3_, LP_4_, LP_5_) and Peptides (P_3_, P_4_, P_5_)

**Figure 3:**
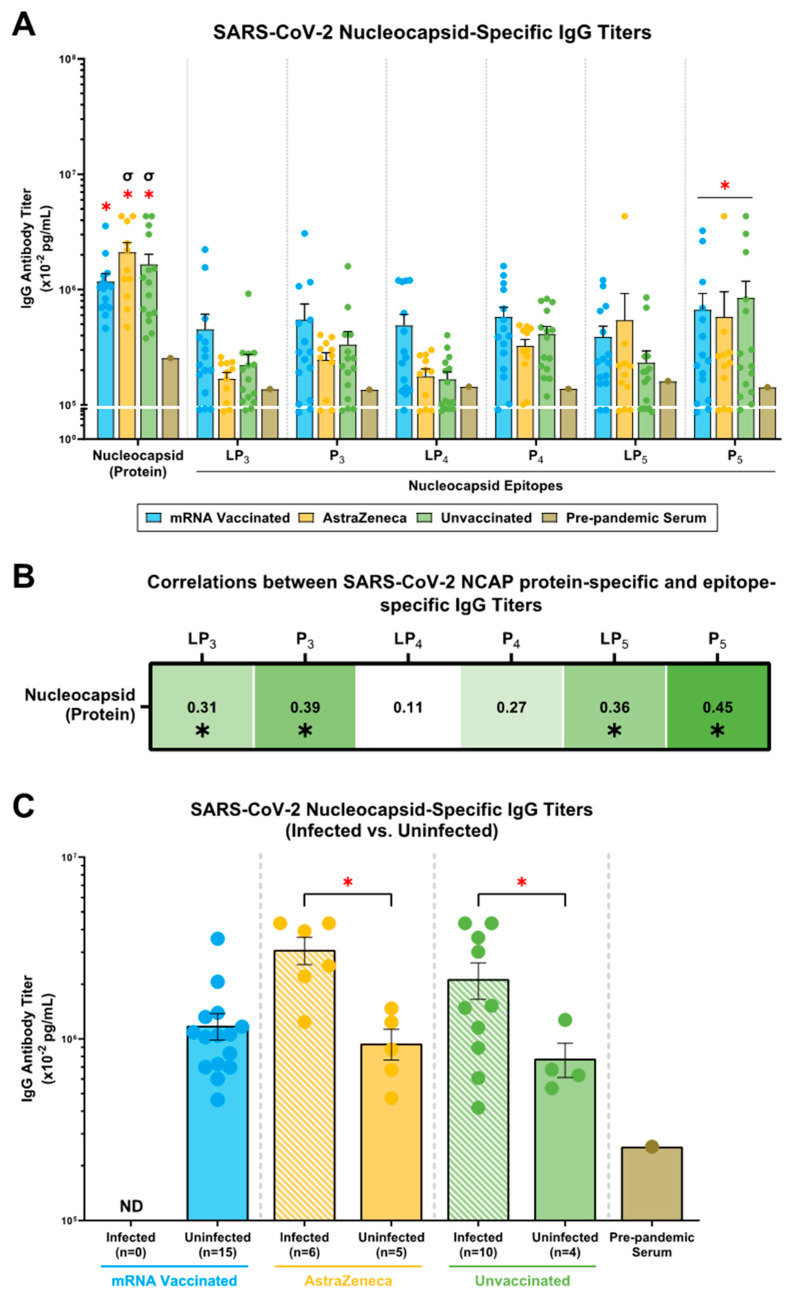
N (protein)-specific and epitope-specific IgG antibody titers in mRNA-vaccinated, AstraZeneca-vaccinated, and unvaccinated donors. (**A**) IgG antibodies against the SARS-CoV-2 N (protein), and N-derived lipopeptides (LP_3–5_) and peptides (P_3–5_) were determined from mRNA-vaccinated (n = 15; blue), AstraZeneca-vaccinated (n = 11; yellow), and unvaccinated (n = 15; green) donors. (**B**) Correlations between SARS-CoV-2 N (protein)-specific and epitope-specific IgG antibody titers were performed. Spearman’s R-values (black) and significant correlations (*p* ≤ 0.05; *) are indicated. (**C**) Within each vaccine group, donors were stratified by known infection with COVID-19 (infected vs. uninfected), and SARS-CoV-2 N-specific IgG antibody titers were shown. Pre-pandemic serum is an experimental control. (**A**,**C**) Bars represent mean ± SEM, with each point representing an individual donor. Statistical significance was determined using a (**A**) two-way ANOVA and (**C**) one-way ANOVA, followed by Tukey’s post hoc test. *, and σ indicates significant differences (*p* ≤ 0.05) between the pre-pandemic serum, the unvaccinated donors, and the mRNA donors, respectively. Abbreviation: ND (not determined).

N (protein)-specific IgG antibody titers were significantly higher in the mRNA, AstraZeneca, and unvaccinated donors compared to the pre-pandemic serum (Figure 3A). Moreover, the AstraZeneca and unvaccinated donors showed higher N-specific IgG antibody titers compared to the mRNA-vaccinated group (Figure 3A). Next, regarding IgG antibody titers against N-derived lipopeptides and peptides, the mRNA-vaccinated group showed the highest titers against LP_3_, P_3_, LP_4_, and P_4_, followed by the unvaccinated and AstraZeneca group (Figure 3A). LP_5_-specific IgG antibody titers were higher in the mRNA and AstraZeneca-vaccinated groups, followed by the unvaccinated donors, which showed similar titers to pre-pandemic serum (Figure 3A). For all donors, the P_5_-specific IgG antibody titers were significantly higher compared to those in pre-pandemic serum; however, similarities were observed among the vaccinated and unvaccinated groups (Figure 3A). Lastly, correlation studies with N (protein)-specific IgG antibody titers showed significant moderate correlations with LP_3_-, P_3_-, LP_5_-, and P_5_-specific IgG antibody titers (Figure 3B). 

Considering the mRNA and AstraZeneca vaccines are spike-based formulations, we hypothesized that N-specific IgG responses were generated through exposure to SARS-CoV-2 during the COVID-19 pandemic. We stratified donors by known infection with COVID-19 and assessed the prevalence of N (protein)-specific IgG antibody titers. For the AstraZeneca and unvaccinated groups, N (protein)-specific IgG antibody titers were significantly higher in donors with known COVID-19 infections compared to donors who did not report an infection (Figure 3C). The mRNA-vaccinated donors did not report any infection, and yet showed a similar N (protein)-specific IgG antibody titer compared to the uninfected AstraZeneca-vaccinated and unvaccinated donors (Figure 3C). Consistent with other studies, these results suggest that N-specific IgG responses in the donor plasma can be attributed to possible prior infection and/or exposure to COVID-19 [24,25,26].

### 2.5. SARS-CoV-2 M-Specific IgG Antibody Responses in mRNA-Vaccinated, AstraZeneca-Vaccinated, and Unvaccinated Donors

**Figure 4:**
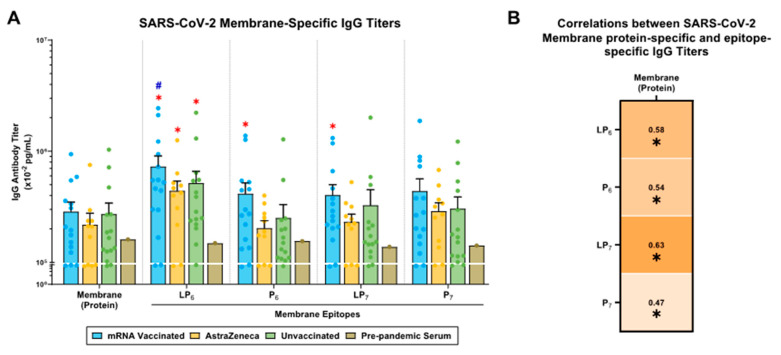
SARS-CoV-2 M-specific IgG antibody responses in mRNA-vaccinated, AstraZeneca-vaccinated, and unvaccinated donors. (**A**) Plasma samples from mRNA-vaccinated (n = 15; blue), AstraZeneca-vaccinated (n = 11; yellow), and unvaccinated (n = 15; green) donors were tested against the SARS-CoV-2 M (protein), and M-derived lipopeptides (LP_6,7_) and peptides (P_6,7_). Pre-pandemic serum was used as control. Bars represent mean ± SEM, with each point representing an individual donor. A two-way ANOVA was used to determine significance, followed by Tukey’s post hoc test. *, and # indicates significant differences (*p* ≤ 0.05) between the pre-pandemic serum, the unvaccinated donors, and the mRNA vs. AstraZeneca donors, respectively. (**B**) Correlations between SARS-CoV-2 M (protein)-specific and epitope-specific IgG antibody titers were performed. Spearman’s R-values (black) and significant correlations (*p* ≤ 0.05; *) are indicated.

In the mRNA-vaccinated, AstraZeneca-vaccinated, and unvaccinated groups, M (protein)-specific IgG titers were higher compared to those in pre-pandemic serum (Figure 4A). All M-derived lipopeptides (LP_6,7_)- and peptides (P_6,7_)-specific IgG responses showed similar titers, with the mRNA-vaccinated donors showing the highest IgG titers, followed by unvaccinated and AstraZeneca-vaccinated donors (Figure 4A). Notably, only the mRNA-vaccinated donors showed significant IgG titers against LP_6_, P_6_, and LP_7_, compared to pre-pandemic serum (Figure 4A). Moreover, the IgG titers in AstraZeneca and unvaccinated donors were significantly higher against LP_6_ compared to those in pre-pandemic serum (Figure 4A). Next, there were strong positive correlations between M (protein)-specific IgG antibody titers with LP_6_-, P_6_-, LP_7_-, and P_7_-specific IgG titers (Figure 4B). 

## 3. Discussion

To develop a broadly protective vaccine that targets multiple SARS-CoV-2 variants, it is important to understand the antigenic homology between SARS-CoV-2, its variants, and other heterologous CoVs. Considering the SARS-CoV-2 S protein, several studies have reported sequence homology between SARS-CoV-2 VOCs (96.23–97.8%), SARS-CoVs (75–80%), MERS-CoVs (>70%), and common cold CoVs (14–30%) [27,28,29]. This sequence identity has contributed to the cross-reactive antibody titers observed in convalescent COVID-19 sera against several SARS-CoV-2 variants and other heterologous CoVs [30,31]. Here, we demonstrate that human plasma samples from mRNA-vaccinated, AstraZeneca-vaccinated, and unvaccinated donors generated cross-reactive IgG antibody titers against the SARS-CoV-2 Alpha (B.1.1.7), Beta (B.1.351), Delta (B.1.617), and Omicron (BA.1, BA.4, BA.5, BQ1.1) variants (Figure 1A). Interestingly, these plasma samples were collected before the emergence of the Omicron lineage (B.1.1.529); therefore, all donors are considered naïve against the tested Omicron variants (BA.1, BA.4, BA.5, and BQ1.1). This indicates that plasma IgG titers against the SARS-CoV-2 Omicron variants (BA.1, BA.4, BA.5, BQ1.1) were originally generated against the Wuhan-Hu-1 strain by the mRNA or AstraZeneca vaccines and/or by natural infection with the Alpha (B.1.1.7), Beta (B.1.351), and/or Delta (B.1.617) variants. This provides evidence of heterologous immunity induced in humans, supporting the idea that immune responses specifically targeted towards one pathogen can mount a response against a novel, never-seen-before pathogen [2]. However, it is important to note that the quantity and/or effectiveness of these cross-reactive antibody responses may not provide sufficient protection against newer SARS-CoV-2 variants, suggesting the need for a next-generation COVID-19 vaccine that enhances protection against multiple variants and prevents SARS-CoV-2 infections.

Conceptually, vaccine-induced and/or naturally acquired heterologous immunity relies on targeting shared epitopes between pathogens [7]. In mRNA-vaccinated, AstraZeneca-vaccinated, and unvaccinated donors, the specificity of the cross-reactive IgG titers significantly correlated with spike-derived epitopes, P_1_ and P_2_, which are structurally and/or sequentially conserved among SARS-CoV-2 VOCs (Figure 1B,C) [23]. Additionally, competitive inhibition of mRNA-vaccinated, AstraZeneca-vaccinated, and unvaccinated plasma samples, with the P_1_ epitope, led to a partial reduction in IgG binding to most of the examined SARS-CoV-2 variants (Figure 2A). Moreover, competitive inhibition with the P_2_ epitope led to a reduction in IgG binding to SARS-CoV-2 spike protein in the unvaccinated group; however, in the mRNA- and AstraZeneca-vaccinated donors, there was an increase in IgG binding (Figure 2B). The addition of P_1_ and P_2_ epitopes served the purpose of competitively inhibiting antibodies to spike proteins in donor plasma samples. However, in the mRNA- and AstraZeneca-vaccinated donors, the addition of P_2_ (epitope) enhanced binding to the spike protein (Figure 2B). Herein, P_2_ may have acted as an agonist for pre-existing IgG antibodies to enhance their binding ability against the SARS-CoV-2 spike antigen. Mouse studies have shown that antibodies induced upon immunization with P_1_ or P_2_ have neutralizing functionality [22]. Therefore, these results provide support for the notion that targeting sequentially conserved regions of the SARS-CoV-2 S-protein induces heterologous (cross-reactive) immunity that recognizes multiple SARS-CoV-2 variants. Applications of heterologous immunity can facilitate the design of next-generation vaccines for SARS-CoV-2, by continual screening for conserved, functional epitopes, such as P_1_ and P_2_, to achieve protection against a broad spectrum of CoVs. Our findings highlight the feasibility of designing a pan-coronavirus vaccine through identifying cross-reactive epitopes among multiple SARS-CoV-2 VOCs and other CoVs to maximize immune coverage against CoVs. 

Current practices of updating the highly variable SARS-CoV-2 spike antigens to account for prevailing variants with repeated boosters is not a long-term solution. Furthermore, regularly updating COVID-19 vaccines may result in immune imprinting and antigenic sin, leading to compromised vaccine efficacy [32,33]. Pre-existing antibodies specific to the Wuhan-Hu-1 strain have been shown to be imprinted and increased with each homologous boost with the BNT162b2 mRNA vaccine [34,35]. Also, imprinting occurs with heterologous boosting by bivalent vaccines that incorporate S-proteins of novel SARS-CoV-2 VOCs [34,35]. Vaccine-induced imprinting results in back-boosting of original humoral immunity while limiting the generation of de novo antibodies against novel immunogens and conserved epitopes. Moreover, administering full-length S-proteins may focus immune responses towards immunodominant, hypervariable regions that promote imprinting, whereas next-generation vaccines need to direct responses toward conserved, cryptic domains, thereby establishing cross-reactivity, while limiting antigenic sin [36].

The majority of the current vaccine efforts against SARS-CoV-2 are limited by targeting the S-protein; however, it is important to consider N and M proteins as potential targets that will allow us to establish cross-reactive responses. Our results demonstrate that mRNA-vaccinated, AstraZeneca-vaccinated, and unvaccinated donors generate N- and M-specific IgG antibody titers. However, within the vaccinated groups, those with known COVID-19 infections showed significantly higher N-specific IgG titer (Figure 3 and Figure 4). Considering that the mRNA and AstraZeneca vaccines are spike-based formulations, the presence of N- and M-specific IgG antibody responses in uninfected donors can be explained by unreported/asymptomatic SARS-CoV-2 infections and/or cross-reactive IgG antibodies generated as a result of prior exposure to human CoVs. Many studies have demonstrated cross-reactive N-/M-specific humoral and cellular responses among common cold CoVs, MERS-CoV, and SARS-CoV that have contributed to sustainable, decade-long immunity in humans [12,15,37,38,39,40]. Previous studies [10,11,12,13,14,15,16,17,18,19,20,21] identified cross-reactive epitopes from the N- (P_3_/P_4_/P_5_) and M- (P_6_/P_7_) proteins that share sequence homology between beta-CoVs, and donor plasma were assayed for IgG against these epitopes. In this study, the N- and M-specific IgG titers showed specificity towards conserved N-derived epitopes (P_3_ and P_5_) and M-derived epitopes (P_6_ and P_7_), respectively (Figure 3 and Figure 4). Furthermore, N-specific antibody titers have correlated with improved clinical outcomes and protection against disease severity. Moreover, these antibodies neutralize viral shedding of the N-protein to restore chemokine function during SARS-CoV-2 infections [9,10,14]. The detection of M-specific antibody titers suggests that there is corresponding cellular immunity targeting the SARS-CoV-2 M antigen [41]. Moreover, antibodies can mediate effector functions, including phagocytosis, opsonization, complement activation, and antibody-dependent cellular cytotoxicity. Therefore, in the quest to find a pan-coronavirus vaccine candidate, targeting non-spike epitopes can facilitate broader coverage of CoVs, expand immune functionality, and provide a multidimensional response against multiple SARS-CoV-2 proteins. 

In conclusion, this study has defined immune cross-reactivity and identified seven epitopes (P_1–7_) from the SARS-CoV-2 S-, N-, and M-proteins that can be further investigated for a multi-valent, pan-coronavirus vaccine candidate. Identification of peptide-epitope specificity of cross-reactive/heterologous humoral responses can serve as a significant milestone in the development of a pan-coronavirus vaccine.

## 4. Materials and Methods

### 4.1. Ethical Approval

The study was approved by the University of Alberta Human Research Ethics Board (HREB; ref. no. Pro0085358).

### 4.2. Human Plasma Donors

Patient samples were acquired from the Canadian BioSample Repository (CBSR), and the pre-pandemic (pooled) serum was collected prior to the COVID-19 pandemic. The serum was commercially purchased (Sigma-Aldrich; St. Louis, MO, USA). 

### 4.3. SARS-CoV-2 Proteins, Lipopeptides, and Peptides

Synthetic lipopeptides (LP_1–7_), peptides (P_1–7_), and SARS-CoV-2 spike, nucleocapsid, and membrane proteins were purchased from Genscript Inc. (Piscataway, NJ, USA), with >96% purity (Table 1) [22]. Spike proteins of the Alpha (B.1.1.7), Beta (B.1.351), Delta (B.1.617), and Omicron (BA.1, BA.4, BA.5, and BQ1.1) variants were obtained. All lipopeptides, peptides, and SARS-CoV-2 proteins were stored at −20 °C and diluted in PBS before use. 

### 4.4. Detection of Human IgG Antibodies and P_1_/P_2_ Competitive Antibody Assays

NUNC MaxiSorp 96-well flat-bottom plates (Thermo Scientific Nunc™; Waltham, MA, USA) were coated with synthetic lipopeptides (LP_1–7_), peptides (P_1–7_), SARS-CoV-2 spike proteins of the Alpha (B.1.1.7), Beta (B.1.351), Delta (B.1.617), and Omicron (BA.1, BA.4, BA.5, and BQ1.1) variants, nucleocapsid, and membrane proteins, in individual plates, at 1 µg/mL in PBS, and incubated overnight at 4 °C. Next, wells were blocked with PBS (1% BSA) for 1h, followed by the addition of plasma samples (1:100; triplicates), which were incubated for 2 h at room temperature (RT). For P_1_/P_2_ competitive ELISAs, diluted plasma samples (1:100) were pre-incubated with either P_1_ or P_2_ peptides (10, 1, 0 µg/mL), at 37 °C for 1 h. Next, the pre-incubated mixtures were transferred to coated plates and incubated for 2 h at RT. Following the incubation, a detection antibody, mouse anti-human IgG Fc-Alkaline Phosphatase (AP; Southern Biotech., Birmingham, AL, USA), was added for 1 h at RT. Subsequently, p-Nitrophenyl Phosphate (PNPP; Southern Biotech., Birmingham, AL, USA) was added, and color development was measured using a DTX 880 Plate Reader (Beckman Coulter, Brea, CA, USA) at 405 nm after 1 h. Using an IgG standard curve, optical density (O.D.) readings were interpolated into [IgG] (pg/mL). Bars are expressed as the mean [Ig] ± SEM of triplicate wells.

### 4.5. Statistical Analyses

Data analysis, statistical analysis, and figures were generated using GraphPad Prism Software 10.2.3 (San Diego, CA, USA). *p* ≤ 0.05 was used to determine significance.

## Figures and Tables

**Table 1:** SARS-CoV-2 derived lipopeptides (LP_1–7_) and their respective peptide (P_1–7_): viral protein, location, code, amino acid sequence, and epitope characteristics.

Pathogen	Protein	Location	Code	Amino Acid Sequence	Epitope Characteristics [10,11,12,13,14,15,16,17,18,19,20,21]
SARS-CoV-2	S_1_	492–505	LP_1_	LQSYGFQPTNGVGYK(Palmitoyl)G	▪ Derived from SARS-CoV-2 Spike S1 subunit ▪ Predicted B cell epitope ▪ Conserved across SARS-CoV-2, MERS, SARS-CoV-1, HCoV-HKU1, -OC43, -NL43, and -229E
			P_1_	LQSYGFQPTNGVGY	
	S_2_	814–826	LP_2_	KRSFIEDLLFNKVK(Palmitoyl)G	▪ Derived from SARS-CoV-2 Spike S2 subunit ▪ Non-RBD epitope for neutralizing antibody ▪ Conserved across SARS-CoV-2 and SAR-CoV-1
			P_2_	KRSFIEDLLFNKV	
	N	358–372	LP_3_	IDAYKTFPPTEPKKDK(Palmitoyl)G	▪ Derived from SARS-CoV-2 Nucleocapsid (N) protein ▪ Predicted CD4+/CD8+ T cell epitope ▪ Binds to multiple MHC I (HLA.A3, A11, A68) and MHC II (HLA-DRB1.01) molecules ▪ Conserved across beta-coronaviruses
			P_3_	IDAYKTFPPTEPKKD	
		317–331	LP_4_	MSRIGMEVTPSGTWLK(Palmitoyl)G	▪ Derived from SARS-CoV-2 Nucleocapsid (N) protein ▪ Predicted CD4+/CD8+ T cell epitope ▪ Binds to multiple MHC I (HLA.A2, A3, A11, A68, B40) and MHC II (HLA-DRB1.01) molecules ▪ Conserved across beta-coronaviruses
			P_4_	MSRIGMEVTPSGTWL	
		158–172	LP_5_	VLQLPQGTTLPKGFYK(Palmitoyl)G	▪ Derived from SARS-CoV-2 Nucleocapsid (N) protein ▪ Predicted CD4+/CD8+ T cell epitope ▪ Binds to multiple MHC I (HLA.A2, A11, A31, A68) and MHC II (HLA-DRB1.01) molecules ▪ Conserved across beta-coronaviruses
			P_5_	VLQLPQGTTLPKGFY	
	M	98–112	LP_6_	ASFRLFARTRSMWSFK(Palmitoyl)G	▪ Derived from SARS-CoV-2 Membrane (M) protein ▪ Binds to multiple MHC II (HLA-DRB1, DRB5, DPA1, DPB1) molecules ▪ Conserved across beta-coronaviruses
			P_6_	ASFRLFARTRSMWSF	
		34–48	LP_7_	LLQFAYANRNRFLYIK(Palmitoyl)G	▪ Derived from SARS-CoV-2 Membrane (M) protein ▪ Conserved across beta-coronaviruses ▪ Binds to multiple MHC II (HLA-DRB1, DRB3, DRB5) molecules
			P_7_	LLQFAYANRNRFLYI	

Note: Adapted from the work of Patel and Agrawal, 2023 [22].

**Table 2:** Demographics of mRNA-vaccinated, AstraZeneca-vaccinated, and unvaccinated donors.

		mRNA Vaccinated (N = 15)	AstraZeneca Vaccinated (N = 11)	Unvaccinated (N = 15)
**Sample Collection Timeline**				
		July 2021–Aug 2021	Apr 2021–Jan 2022	July 2020–July 2021
**Sex, n (%)**				
	**Females**	11 (73.3%)	6 (54.5%)	6 (40.0%)
	**Males**	4 (26.7%)	5 (45.5%)	9 (60.0%)
**Age, median (range)**				
		25 (19–65)	58 (28–63)	42 (20–94)
**Reported COVID-19 Infection, n (%)**				
		-	6 (54.5%)	10 (66.6%)

## Data Availability

The data supporting the findings in this article will be made available by the authors, without undue reservation, upon reasonable request.
